# Classic Kaposi sarcoma: Diagnostics, treatment modalities, and genetic implications – A review of the literature

**DOI:** 10.2340/1651-226X.2024.40537

**Published:** 2024-10-16

**Authors:** Ron Batash, Alberto Crimí, Riad Kassem, Murad Asali, Ishay Ostfeld, Carlo Biz, Pietro Ruggieri, Moshe Schaffer

**Affiliations:** a Orthopedics and Orthopedic Oncology, Department of Surgery, Oncology and Gastroenterology DiSCOG, University of Padova, Padova, Italy; b Dermatology Unit, Sheba Medical Center, Ramat-gan, Israel; c Tel Aviv University, Faculty of Medicine, Tel Aviv, Israel; d Urology Department, Barziali Medical Center, Ashkelon, Israel; e Faculty of Health Sciences, Ben Gurion University of the Negev, Israel; f Department of Thoracic Surgeon, Baruch Padeh Medical Center, Poriya, Israel; g Oncology Department, Barzilai Medical Center, Ashkelon, Israel

**Keywords:** Classic Kaposi sarcoma, treatment, guidelines, gene mutations, BPTF

## Abstract

**Background and purpose:**

Classic Kaposi sarcoma (CKS) is a rare vascular disease mainly found in populations of Mediterranean origin. The pathogenesis involves Human Herpes Virus 8 (HHV8) and genetic mutations such as SNP309 in the MDM2 gene. The recently discovered BPTF mutation in cells of CKS patients demonstrated higher latency-associated nuclear antigen (LANA) staining and altered vital transcriptomics, implicating a potential role in tumorigenesis. This review explores the genetic underpinnings and treatments for CKS.

**Material and methods:**

A comprehensive literature search was conducted from 2004 to 2024, yielding 70 relevant papers. Ongoing clinical trials investigating novel treatments such as talimogene and abemaciclib were included in the search and presented in the results.

**Results:**

Clinical diagnosis and treatment can be challenging as the number of studies on CKS and treatment modalities is limited. Treatment strategies vary by disease stage, with local therapies like surgical intervention and radiation therapy recommended for early stages, while systemic therapies are considered in cases of systemic disease.

**Interpretation:**

While advancements in CKS treatment offer hope, further studies on immunotherapy are warranted to broaden the therapeutic options, such as anti-bromodomain or BPTF-targeted therapy.

## Introduction

Kaposi sarcoma, first described by Moritz Kaposi in 1872, is considered a rare vascular pathology affecting 1:100 000 patients per year with a male-to-female ratio of 2:1 and 5:1 in Italy and Israel, respectively [1, 2]. Based on other publications, cases of Kaposi sarcoma (KS) subclusters were found in Druze, Cypriot Greek, and Jewish populations [3].

The lesion, arising from lymph nodes or vascular endothelial cells, is further categorized into four subtypes: the Endemic type, found mainly in Africa and occurring in children; the Classic type, in populations of Mediterranean and Eastern European descent, occurring in Elderly immunocompetent men; acquired immune deficiency syndrome (HIV associated); and iatrogenic after immunosuppression therapy, for example, calcineurin inhibitors in cases of organ-transplant patients [4].

Among the known risk factors for developing classic Kaposi sarcoma (CKS) is previous infection of Human Herpes Virus 8 HHV8 (Kaposi sarcoma-associated Herpes virus), age 50 and above, contact with silicaceous volcanic soil and bloodsucking insect bites [5, 6].

Rare familial cases of CKS have been reported in the literature, suggesting a potential genetic predisposition. Associations have been proposed between CKS and various HLA alleles, as well as genetic variants in IL6, IL13, STAT4, and ILRB8. Additionally, STIM1 deficiency has been shown to adversely affect the immune system, potentially leading to a more aggressive manifestation of CKS. A recent study further supports this by demonstrating that BPTF-mutated cells exhibit increased latency-associated nuclear antigen (LANA) staining and significant alterations in critical transcriptomic profiles, highlighting a possible role in tumorigenesis [7–10].

The clinical manifestations of CKS can be seen as asymptomatic bruises in the lower extremities, purple to reddish-brown macules, or lymph node enlargement with the potential of visceral organ involvement. Due to the rarity of CKS, the lesions might be misdiagnosed as arterial insufficiency or venous stasis [11]. Up to 33% of the patients will develop a secondary primary malignancy such as non-Hodgkin lymphoma [12].

The treatment of CKS is varied depending on the stage of the disease. For localized disease, radiation therapy, surgical resection, cryotherapy, and topical therapy are suggested, while for advanced disease, radiation therapy, biological and targeted therapy, chemotherapy, and immunotherapy should be considered [2].

The purpose of this paper is to review the current updates in the genetic and treatment options for CKS. A complete thorough mechanism of action of each therapy presented in this paper is beyond the scope of this review.

## Material and methods

According to PRISMA guidelines, two independent investigators searched the main scientific online databases (Cochrane, PubMed, Google Scholar, Web of Science) and Clinical Trials Registries (USA Clinical Trial Registry, EU Clinical Trials Register, Japan Registry of Clinical Trials) with a combination of Key Words: ‘Classic Kaposi Sarcoma’ AND ‘genetic pathways’ AND ‘treatment’ AND ‘clinical trials’ in the time frame 2004–2024.

Our study was focused exclusively on CKS. Therefore, our inclusion criteria encompassed studies on CKS, including reviews and clinical trials, and ongoing clinical trials investigating molecular and genetic pathways specific to CKS patients. We excluded studies related to non-CKS or secondary Kaposi sarcoma (HIV associated) from our search.

The initial search included 574 papers; after screening, cross-checking, and abstract readings, 151 articles were selected. The two main investigators narrowed down the selection after reading the full articles, omitting the repetitions, omitting irrelevant studies to the topic in question, and selected to 70 papers.

## Results

### Pathogenesis

HHV8, a DNA oncogenic virus, is found in more than 95% of KS patients, including the classic type. The pathogen can be found in the saliva and semen and can be transmitted via fluid contact and sexual intercourse [12, 13]. Being an oncogenic virus, HHV8 interferes with the pathways of P53 in the following ways: 1. The ORF 73 gene, which is responsible for LANA and found on the HHV8 genome, interferes with p53 transcription and activation that inhibit the ability of apoptosis [14]. 2. The ORFK10/K10.1 gene, which encodes the viral interferon regulator factor 4 (VIRF4) found on the HHV8, interferes with the regulation of p53 via proteasome-mediated degradation that interacts with the murine double minute 2 (MDM2) human homologue. This leads to a reduction in p53 levels and suppression of apoptosis [15].

Several studies demonstrated the single-nucleotide polymorphism 309 (SNP309) variation in the MDM2 gene, which results in an increase of the MDM2 protein due to the affinity to the transcriptional activator Sp1 to inactivate p53-MDM2 complexes. This polymorphism was considered associated with breast cancer, soft tissue sarcoma, diffuse large B-cell lymphoma, and nonsmall cell lung carcinoma, and CKS [16, 17]. In the case of Kaposi sarcoma, patients infected with HHV8 were more susceptible to developing visceral Kaposi sarcoma, multicentric Castelman’s disease, or primary effusion lymphoma [2, 18].

In a recent publication, the inherited missense mutation in BPTF associated with dysmorphism and limb anomalies in a neurodevelopmental syndrome [19] was found in CKS patients whose cells demonstrated higher LANA staining, latent phenotype in viral transcriptomics, decreased virion production, and higher latent-to-lytic ratio. The p53 pathways, IFI16, SHFL HLA, TGFB1, and HSPA5 were downregulated [8].

BPTF interacts with ORD73-LANA protein [20], and according to Yogev et al. [8], higher expression of LANA was seen in BPTFT/T cells compared to wild-type cells. In addition, propagation of KSHV in its latent state showed an increase in the infected cell population due to the increased tethering of the LANA-BPTFT/T complex to a sister chromatid. Another suggested mechanism is that the increased tumorigenicity of BPTFT/T is the gain-of-function effect by the virus and the loss-of-function effect in the host. Hence, the combination of virus-host interactions, BPTFI2012T mutation, immune function, and proliferation of the virus contributes to the formation of CKS.

### Diagnosis

The diagnosis of Kaposi sarcoma is based on the clinical suspicion of symptomatic patients and verified by a skin punch or excisional biopsy, which is pivotal to rule out HIV-related KS. Histologically, the characteristic features are spindle cells, vascular proliferation, and positive immune histochemistry for LANA1, CD34, PECAM-1, D2-40, BCL-2, VEGFR-3, and factor VIII [21, 22].

### Staging

There is no consensus on CKS staging classification. Brambilla et al. [23] introduced a valid staging system for CKS in 2003. It is divided into four stages based on the progression and spread of the disease observed in 300 patients with CKS: stage 1, maculo-nodules on the lower extremity; stage 2, infiltrative stage, plaques and sometimes small nodules are seen on the lower extremities; stage 3, florid stage, ulcerated multiple angiomatous plaques and nodules; and stage 4, the disseminated stage, multiple plaques and angiomatous nodules extending beyond the lower extremities. Staging is performed with a total-body CT, mainly to identify stages 3 and 4 [24].

### Treatment

The treatment for Kaposi sarcoma is determined by the stage of the disease (Table 1). Yet, the treatment strategies are not well established due to the low number of randomized clinical trials on CKS. In cases of local disease, the recommended treatment is observation or local therapy via surgical removal or radiotherapy, while systemic therapy is considered in cases of rapidly progressive or metastatic disease [23, 25].

**Table 1:** Treatment options for Kaposi sarcoma, summary table.

Kaposi Sarcoma Staging	Treatment Options	Treatment Subtypes
Stage I, Stage II (Local Disease)	Local Therapy	-Local Treatments: Cryotherapy, CO_2_-laser, Nd:YAG laser, Imiquimod, Timolol, Alirtretinoin gel
		-Intralesional Chemotherapy (Vinblastine, Bleomycin)
		-Brachytherapy
		-Surgery (mainly diagnostic purpose)
Stage III (Progressive) Stage IV (Metastatic)	Systemic Therapy	- Chemotherapy: Doxorubicin (first-line treatment), Paclitaxel
		- Antiangiogenic agents: pomalidomide, lenalidomide, bevacizumab
		- Immunotherapy: Nivolumab, Ipilimumab
		- Other treatments: Interferon alfa-2b, Talimogene, Abemaciclib, Immuno-cytokine NHS-IL12, BPTF gene mutation targeted therapy (future?)
		- Radiation Therapy (for visceral lesions when systemic treatment not possible)

### Local treatments

#### Topical treatment

In a study by Odyakmaz et al. [26], Imiquimod was compared with cryotherapy to treat cutaneous lesions in CKS. A complete response was seen in 42.3% of lesions treated with Imiquimod (vs. 50% in lesions treated with cryotherapy), while a partial response in 73.1% of lesions treated with Imiquimod (vs. 87.5% treated with cryotherapy). Statistically, there was no significant difference between the two treatments. An older study showed a complete response in 47% of patients with classic KS cutaneous lesions [27]. Timolol 0.1% topical gel, a beta blocker that affects KS lesions by VEGF downregulation, demonstrated complete response in 61.5% of patients (no response in 7.7%). According to a recent review, the use of Alitretinoin gel 0.1% (Cis-retinoic acid), a known treatment for its antitumor effect due to the transcriptional regulation to inhibit cell proliferation and apoptosis [28] for the treatment of KS cutaneous lesions, had a partial response rate up to 83.5%; a complete response was found only in 1% of patients [29].

#### Radiation therapy

KS has a marked radiosensitivity; the preferred radiation modality is brachytherapy, with a relatively low dose and no systemic side effects. According to Aral et al. [30], the local response rate ranges between 47% and 90% [30]. Radiation therapy also showed its efficacy in the treatment of KS visceral lesions. Localized lesions can be treated with 30 Gy fractioned in 2–3 Gy doses, while visceral lesions should be treated with 40 Gy. Complications are postradiation erythema and fibrosis; there is a low risk of secondary skin cancers [31–33].

#### Surgical treatment

Surgery has a limited role in the treatment of Kaposi sarcoma due to its nature as a systemic disease with possible multiple sites involved. According to the Association of Scientific Medical Societies in Germany (AWMF) guidelines, the role of surgical therapy can be considered as a palliative therapy or for cosmetic purposes [34]. However, it can be used as a tool for initial diagnosis (by excisional biopsy) and as a primary treatment in small, well limited, and superficial lesions, with the burden of high risk of local recurrence [35]. The risk of functional impairment and the requirement of demolitive surgeries should be taken into consideration when treating large lesions and recurrent disease [36].

#### Cryotherapy and laser

Local cryotherapy and CO_2_ laser are effective and safe treatments in small and superficial lesions, with a local response rate between 63% and 90% and without major complications. Minor complications were scarring, depigmentation of the skin, and blistering [26, 37]. Özdemir et al. demonstrated a local control with 100% response for CKS using a long-pulsed Nd:YAG laser, which emits a wavelength of 1.064 nm to allow penetration into vascular structures and induces minimal damage to the surrounding tissue [38].

#### Local or intralesional chemical or immune-modifying agents

Intralesional chemotherapy brings a high concentration of chemotherapy agent to the site of the lesion with a low systemic dose (and thus reduced side effects). Vinblastine was used as intralesional chemotherapy for treating CKS with a good response rate, between 70% and 98.7% [39, 40]. Bleomycin, used in combination with electroporation (electrochemotherapy), permitting an even higher local concentration and lower systemic dose, showed a complete response between 65% and 89% of cases [41, 42].

### Systemic treatments

#### Doxorubicin

Doxorubicin is the first-line systemic treatment for Kaposi sarcoma. It is administered intravenously as pegylated-liposomal doxorubicin (PLD) every 3 weeks with a 20 mg/m^2^ dose. Its efficacy was demonstrated in treating HIV-related KS, classic, and transplant-associated KS [43, 44]. In randomized trials comparing PLD with doxorubicin/bleomycin/vincristine association [45] and bleomycin/vincristine association [46], PLD showed a statistically significant higher response rate (46% vs. 25% in the first trial and 59% vs. 23% in the second) [45, 46], with a time to treatment failure of 4 months and a duration of response of 25 months [47]. The overall response rate in the treatment of classic KS was between 71% and 100% [47], with a time to treatment failure of 4 months and a duration of response of 25 months [47]. The overall response rate in the treatment of CKS was between 71% and 100% [47].

Side effects of the treatment with PLD were nausea and vomiting, anemia, leukopenia, and peripheral neuropathy. Moreover, severe neutropenia was seen in 5% of cases, and severe hand-feet syndrome was seen in 5% of patients [47–50].

#### Paclitaxel

Paclitaxel is considered another first-line therapy for CKS and is administered at a dose of 100mg/m^2^ every 14 days with effective results up to 60% remission [2, 51]. In a study published in 2023 by Paksoy et al. [52], the effectiveness and safety of 80–100 mg administered intravenously weekly were evaluated. Their results showed complete response, partial response, and stable disease in 15.9%, 63.7%, and 13.6% of their patients, respectively. Neutropenia was seen in 9.1% of the cases, and peripheral neuropathy was seen in 6.8% [52]. In other studies, paclitaxel was more myelotoxic, with alopecia, onycholysis, arthralgias, myalgias, and chronic fatigue syndrome documented. According to the German S1 Guidelines for the management of Kaposi sarcoma, the therapy may benefit patients who have received prior chemotherapy or have had progressive disease [53].

#### Antiangiogenic agents (pomalidomide/lenalidomide/bevacizumab)

The overexpression of angiogenetic factors in KS can be targeted by various treatments [54]. The most recent one is pazopanib, a tyrosine kinase inhibitor that inhibits VEGF receptors (already used for treating renal cell carcinoma and metastatic soft tissue sarcomas). Only one case is described in the scientific literature of a CKS patient treated with Pazopanib [55].

Thalidomide and its analogs pomalidomide and lenalidomide have anti-angiogenetic capacity, inhibiting VEGF production [56]. Pomalidomide was tested on 10 patients with CKS in a trial [57] in 2022; there was a response in 80% of the patients. The FDA has approved pomalidomide for treatment of KS.

Bevacizumab is an anti-VEGF monoclonal antibody used in lung, breast, and GI tract cancers. It was tested in a phase II study in KS, showing a 31% response rate [58], while in another study, it did not have beneficial effects [59].

Imatinib targets PDGF and c-KIT receptors involved in KS development. In a phase II study, it had a response rate of 29% with a high rate of toxicity [60].

#### Immunotherapy

Immunotherapy targeting immune response checkpoints was found effective in other sarcoma types. The mechanism of action is complex and involves multiple pathways, resulting in the inhibition of the host immune response and anticancer mechanism. In a recent study [25], CKS was treated with 240 mg of Nivolumab (every 2 weeks) and 1 mg/kg every 6 weeks of Ipilimumab. The 18 patients in this study had a 62% complete response rate (evaluated with PET-CT). Adverse reactions were noted in 22% of patients; the most severe complications were colitis, pneumonitis, and lipase elevation.

## Discussion

Classic Kaposi sarcoma affects 1:100 000 patients per year, predominantly male. While the disease is mainly found among central and eastern European Jews, Schaffer et al. [61] found an earlier onset of the disease in a non-Jewish population of the Druze at the west Galilee in Israel and a higher rate of appearance among non-Ashkenazi Jews, mainly Sephardi-origin Jews. It is also known that Cyprotic Greek, Jewish, and other Mediterranean populations spread apart in diaspora processes maintained a higher incidence of CKS, pointing out the genetic implications of the disease [3].

It is a challenging pathology for diagnosis and treatment; multiple therapeutic modalities have been developed in the past 20 years. Based on European and United States guidelines, local therapy should be considered for Stage I and II disease (local disease), while systemic therapy should be reserved for Stage III progressive and Stage IV metastatic (or visceral) disease (Table 1).

According to National Comprehensive Cancer Network (NCCN) guidelines 2024 [62], a cutaneous staged disease in an asymptomatic patient with an acceptable cosmetic lesion may be considered for observation only, while a symptomatic patient should undergo local treatment comprising, as described in the above treatment section, local excision, and toxic therapy or radiation therapy to control the disease and reduce the recurrence rate. Our search found that radiation therapy demonstrated up to 90% response in conjunction with local therapy.

In a patient with an advanced stage, Doxorubicin is considered the first-line therapy. There is a risk of severe lymphedema and delayed wound healing after radiation therapy. Therefore, in cases of a severe stage of the disease, it is preferable to avoid the conjunction of radiation therapy with systemic therapy. Radiation therapy should be considered when systemic therapy is not feasible, for example, short-term disease management as a bridging treatment for systemic therapy.

**Figure 1:**
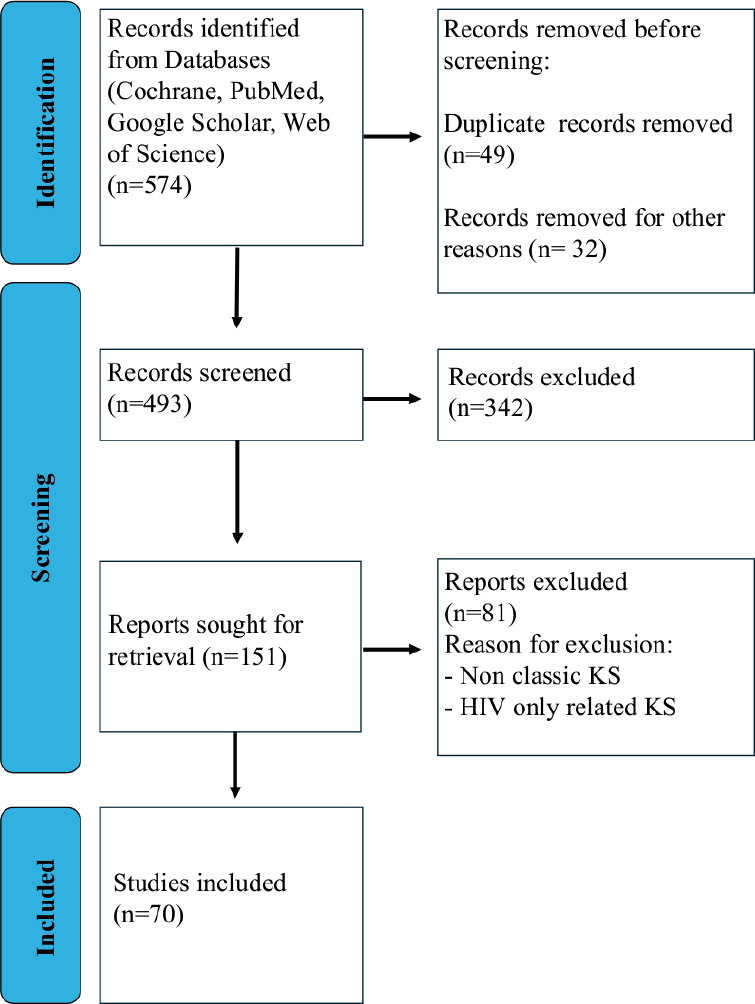
PRISMA algorithm search flowchart.

This review is focused on CKS and presents an up-to-date literature search up to March 2024. The search was performed on primary scientific databases according to PRISMA guidelines (Figure 1**)** and on clinical trial registries available (Table 2). In our search of the literature, the number of classic KS patients and prospective studies was low, while most of the studies on KS were HHV-8 or HIV related. In our search, we excluded HIV-related KS treatments and focused on the sub-type of classic KS only.

**Table 2:** Ongoing and future clinical trials on Classic Kaposi Sarcoma (updated on the 7th of March 2024).

Drug tested	Study ID	Phase and description	Mechanism of action
Pembrolizumab Lenvatinib	MK-3475-B60	Phase II – relapsed/refractory Classic Kaposi Sarcoma (CKS)	Pembrolizumab is a monocolonal antibody that inhibits lymphocytes PD-1 receptors. Lenvatinib is a receptor tyrosine kinase (RTK) inhibitor, it inhibits the kinase activities of vascular endothelial growth factor (VEGF) receptors
Talimogene Laherparepvec	P17072019	Phase II – Multicenter Study in Classic or Endemic Kaposi Sarcoma (KAPVEC)	Oncolytic immunotherapy (used for unresectable melanoma) PD-1/PD-L1 axis inhibitor
Abemaciclib	NCT04941274	Phase I and II – Abemaciclib in patients with HIV-associated and HIV-negative Kaposi Sarcoma	Cyclin-dependent kinase (CDK) inhibitor targeting CDK4 and CDK6 cell cycle pathways
NHS-IL12 M7824	NCT04303117	Phase I and II – NHS-IL12 Monotherapy and in combination with M7824 in Advanced Kaposi Sarcoma	Targets the exposed DNA of the necrotic tumor with local immune response
Doxorubicin Pomalidomide	NCT02659930	Phase I – Pomalidomide in combination with Liposomal Doxorubicin in people with advanced or refractory Kaposi Sarcoma	Doxorubicin is a cytotoxic agent and Pomalidomide has anti-angiogenetic capacity, inhibiting VEGF production
Pomalidomide	NCT04577755	Phase II – Pomalidomide Treatment in patients with Kaposi Sarcoma	Pomalidomide has anti-angiogenetic capacity, inhibiting VEGF production
Human Antibody-Cytokine Fusion	2016-003239-38	Phase III – study comparing the efficacy of the combination of doxorubicin and the tumor-targeting human antibody-cytokine fusion protein L19TNF to doxorubicin alone as first-line therapy	Human antibody-cytokine fusion protein L19TNF is an immune-cytokine, an antibody-based delivery of pro-inflammatory payloads (such as IL2, IL12, and TNF) to the tumor microenvironment

In a recent publication [8], a new possible treatment pathway was suggested based on the genetic mutation of the BPTF gene found in CKS. The role of BPTF gene mutation (connected to the p53 mechanism) in tumor progression has been found in other cancer types, for example, lung adenocarcinoma, glioma, breast carcinoma, and vascular-based tumors. This mutation can be found in vascular-based tumors and in CKS.

According to de Oliveira et al. [63] and Küppers et al. [64], the activation and deregulation of the NF-κB complex is a critical feature in the pathogenesis of KSHV, with similar features to EBV-induced tumorigenesis in Reed Sternberg tumor cells of Hodgkin lymphoma. Yogev et al. demonstrated the effects of the Rel–NF-κB complex on the ratio of infected BPTFT/T and BPTFwt, hence regulating the oncogenesis [8]. In the same study, the role of BPTF in tumorigenesis was demonstrated, and the authors suggested that it might be a target for novel immunotherapy, such as anti-bromodomain or BPTF-targeted therapy.

Recent publications [65, 66] have identified several immunotherapeutic mechanisms of action in the treatment of sarcomas, including checkpoint blockade (e.g. CTLA-4 inhibitors like Ipilimumab and PD-1 or PD-L1 inhibitors such as Nivolumab and Avelumab), oncolytic viruses, cancer-targeted antibodies, vaccine therapy, and adoptive cell therapies like TCR therapy. These treatment modalities underscore the role of immunotherapy in targeting genetic mutations, which may provide insights into ongoing clinical trials and their effects on CKS.

Another treatment for KS is Interferon alfa-2b. It was used in clinical trials for treatment of HIV-related KS, while for classic KS there is only a 1996 Phase II clinical trial on 16 patients, with a major response documented in 10 patients [67].

Among the recently investigated treatments, we found that Talimogene, an oncolytic immunotherapy (used for unresectable melanoma) PD-1/PD-L1 axis inhibitor, showed efficacy in treating Merkel cell carcinoma (a virus-induced cancer, similar to KS) and is now a target for a phase II clinical trial in classic KS.

Abemaciclib is a cyclin-dependent kinase (CDK) inhibitor approved for metastatic breast carcinoma patients. It stops the cell cycle by targeting CDK4 and CDK6 cell cycle pathways. It is now in ongoing phase I and II studies for non-HIV Kaposi sarcoma.

There is an ongoing trial (NCT04303117) on the immuno-cytokine NHS-IL12 (M9241), which targets the exposed DNA of the necrotic tumor with a local immune response without high systemic toxicity and M7824, a fusion protein, in the treatment of classic KS. According to the investigators, similar drugs showed a good response in other virus-related tumors.

In conclusion, the different modalities of treatment described above and listed in the table might expand the treatment options to increase the overall response and reduce recurrence rates. However, they are still experimental protocols and need phase III trials to confirm their efficacy.

Studies on new treatments for CKS are required and will enrich the scientific literature in the field and improve the ability to deal with this disease.

## Author contributions

Conceptualization, R.B., A.C., and R.K.; methodology, M.S.; validation, I.O., C.B., and P.R.; formal analysis, M.A.; investigation, R.B., A.C., and R.K.; writing—original draft preparation, R.B., A.C., and R.K.; supervision, P.R. and M.S. All authors have read and agreed to the published version of the manuscript.

## Data Availability

The data that support the findings of this study are available from the corresponding author, A.C., upon reasonable request.
